# Explaining Variation in the Percentage of Admissions for Patients Aged 80 and Over in Municipal InPatient Acute Care Services: A Cross-Sectional Analysis From Norway

**DOI:** 10.1177/11786329261490712

**Published:** 2026-09-22

**Authors:** Hanne Marie Rostad, Marianne Sundlisæter Skinner, Lisa Victoria Burrell, Fan Yang, Trond Tjerbo, Torunn Kitty Vatnøy

**Affiliations:** 1 Department of Health Sciences, Center for Care Research, East, The Norwegian University of Science and Technology, Gjøvik, Norway; 2 Department of Health Management and Health Economics, University of Oslo, Oslo, Norway; 3 Department of Health and Nursing Science, University of Agder, Grimstad, Norway

**Keywords:** admission avoidance, health services research, intermediate care, municipal inpatient acute care, older adults

## Abstract

**Background:**

Municipal Inpatient Acute Care (MIPAC) units in Norway provide short-term inpatient services, but structural and contextual disparities, spanning resources, competence, and accessibility, may affect utilization patterns.

**Objectives:**

To examine to what extent variation in the percentage of admissions involving patients aged ≥80 years across MIPAC units is associated with structural and contextual conditions.

**Design:**

Cross-sectional study using aggregated register and survey data.

**Methods:**

The analytical sample included 147 MIPAC units, corresponding to 68% of the 215 units in the administrative dataset. Descriptive statistics describe sample characteristics and admissions by age group. Pearson correlations assessed interrelationships and multicollinearity before regression analysis.

**Results:**

The model explained 38% of the variance. Unit location, registered nurse coverage, and driving distance to the nearest emergency department were associated with the percentage of admissions involving patients aged ≥80 years. Units located in GP out-of-hours clinics had a lower percentage compared to nursing home–based units (B = -17.12, 95% CI [-23.51 to -10.73], β =-0.41, p < 0.001). Greater registered nurse coverage (B = -0.12, 95% CI [-0.22 to -0.02], β = -0.18, p = 0.021) and longer driving distance to the nearest emergency department (B = -0.07, 95% CI [-0.11 to -0.04], β = -0.31, p < 0.001) were also associated with lower percentages. The estimate for the proportion of adults ≥80 years in the MIPAC catchment area was positive, but uncertain, as the confidence interval included zero (B = 1.76, 95% CI [-0.06 to 3.57], β = 0.20, p = 0.057).

**Conclusion:**

MIPAC admission patterns for the oldest patients were associated with both service configuration and local context. These findings highlight variation in the use of MIPAC services across different units. Future research should examine how structural and contextual factors are related to patterns of MIPAC utilization and explore the mechanisms underlying these associations.

## Introduction

Nationally and internationally, reducing emergency hospital admissions has become an increasingly urgent priority. This is driven by the need to ensure sustainable use of healthcare resources, reduce escalating healthcare costs, and reduce the risks associated with hospitalization.^1-3^ Reducing hospital-related risks is particularly important for older adults due to their increased vulnerability to complications and psychological stress during hospitalization. Studies have shown that older patients are more likely to experience complications unrelated to the condition for which they were admitted, and many are discharged with reduced ability to perform daily activities compared to their baseline.^4,5^ Hospitalization of older adults is associated with a heightened risk of adverse events, such as hospital-acquired infections, delirium, falls, pressure ulcers, venous thromboembolism, functional decline, and increased mortality.^5,6^ These risks have led to a growing consensus that avoidable hospital admissions among older adults should be minimized whenever possible.^3^ Reducing such admissions not only alleviates individual distress and improves quality of life, but also significantly lowers the financial burden associated with prolonged hospital stays, readmissions, and complex care needs.^1^ As a result, targeted strategies and initiatives to prevent avoidable hospital admissions in older populations are essential to improving patient outcomes, but also for ensuring the long-term sustainability of healthcare systems.

In Norway, one such initiative is the Municipal In-Patient Acute Care (MIPAC) service, which was introduced as part of the 2012 Coordination Reform.^7^ MIPAC is a form of admission avoidance intermediate care scheme, established as an alternative to hospital admission, aiming to reduce pressure on specialist health services by offering patients timely care closer to home. MIPAC provides short-term, in-patient treatment at the municipal level, and since its inception, MIPAC has primarily served older patients. In 2022, only 28 percent of admissions nationwide involved patients under the age of 67.^8^

Responsibility for implementing MIPAC was delegated to individual municipalities, resulting in considerable variation in how the service has developed across the country. Given the wide range in municipality size—from fewer than 300 to approximately 700,000 inhabitants—the scale and organization of MIPAC services differ substantially. Some municipalities operate units with only a fraction of a MIPAC bed (e.g., 0.2 of a nursing home bed), while others run facilities with up to 72 beds. MIPAC units may be physically located in different types of facilities, i.e., nursing homes, general practitioner (GP) out-of-hours clinics, intermediate care institutions, or other municipal facilities, depending on local infrastructure and resources. Norway’s varied geography—with many rural and peripheral areas—also poses challenges related to long travel distances to hospitals and MIPAC units. This has contributed to substantial variation in both how MIPAC is organized^9^ and how it is used.^10,11^ Previous qualitative studies have identified barriers to use, including limited trust in staff competencies,^12,13^ and have highlighted the need for increased physician presence and higher nursing competence.^9,14^ Quantitative studies have examined aspects of patient flow and service utilization within municipal acute inpatient services. Most of this work has been based on individual units or small regional samples,^15-17^ offering insights into diagnostic needs, clinical presentation, and transfer patterns. One more recent study has used national-level register data to analyze variation in admission sources and discharge destinations across MIPAC units.^18^ Taken together, previous studies on MIPAC services contribute to our understanding of variation in service use, but they provide limited evidence on how structural and contextual conditions shape differences across units.

Norwegian municipalities face growing challenges in financing and staffing health and care services, including MIPAC. These challenges stem from rising primary health care costs, declining municipal revenues^19^ and persistent difficulties in recruiting and retaining qualified personnel.^20,21^ As a result, municipalities differ in how the MIPAC service is organized and resourced, creating structural and contextual conditions at a service level that may influence patterns of utilization. Evidence shows that the volume and scope of MIPAC services have been adjusted over time in response to local needs, resource constraints, and patterns of service utilization.^22^ Consequently, the capacity to deliver consistent and high-quality services varies across MIPAC services, reflecting differences in professional competence, available resources, and local priorities.^21^ Understanding these patterns is important because structural and contextual variations in how MIPAC services are used may be related to differences in care pathways and resource allocation. Such differences may have implications for patient safety, care quality, and the overall effectiveness of the healthcare system. This is particularly critical for the oldest old (80+), who often have complex needs and limited capacity to navigate fragmented services. Understanding whether and how local conditions are linked to service utilization may provide a basis for further research on equitable and coordinated care for this group.

Against this backdrop, this paper examines the extent to which variation in the percentage of admissions involving patients aged 80 years and older is associated with structural and contextual conditions. Structural conditions refer to internal characteristics of the MIPAC units, including organization, staffing, and capacity—typically stable and shaped by local policies and resources. Contextual conditions encompass external factors such as municipal geography, demographics, and administrative affiliation, which are often beyond the unit’s direct control.

## Methods

We employed a cross-sectional research design to examine the extent to which variation in the use of MIPAC services for patients aged 80 years and older is associated with organizational and contextual characteristics. The analysis combines three sources of data, all structured at the MIPAC unit level: registry data on MIPAC services, contextual data originally collected at the municipal level, and survey data on physician and nursing coverage within MIPAC units to capture variation in staffing. The survey data were collected as part of a study conducted at the University of Agder and are included here as one of the data sources for the present analysis. The survey represented point-in-time data collection, and all contextual data reflected conditions in the same calendar year. The study follows the Strengthening the Reporting of Observational Studies in Epidemiology (STROBE) guidelines for cross-sectional studies.

### Data Sources

All variables used in the analysis were based on data from 2019. The choice of 2019 was based on two main considerations. First, it represents the most complete and reliable dataset available from the Directorate of Health, which has required all municipalities to report MIPAC admissions and administrative data since 2016. Second, it was the year that a national online survey^21^ was conducted. Admissions data from 2017-2019 and 2021-2022 show that the age distribution of patients receiving MIPAC services remained relatively stable throughout this period, with only minor year-to-year variation.^23^ This consistency supports the use of 2019 as a representative year for understanding service use patterns, while avoiding potential confounding effects related to the COVID-19 pandemic.

All data were aggregated at the MIPAC unit level, which corresponds to either a single municipality or a group of municipalities collaborating to deliver the service. In some cases, municipalities had multiple MIPAC units due to their large populations and/or extensive geographical areas. These units were combined into a single entry per municipality to maintain consistency in the unit of analysis in our study.

Data on MIPAC admissions, geographic distribution, and bed capacity were obtained from the Norwegian Directorate of Health’s administrative dataset on MIPAC units^23^ (please see *Data availability* section of this paper for direct links). These data were collected via a national survey conducted by the Directorate, targeting all operational MIPAC units. The resulting dataset included information from 215 units, covering 418 municipalities—representing approximately 99% of the total municipal population in Norway. Data on regional health authority affiliation, the percentage of residents aged 80 years and above in the MIPAC catchment area, and driving distance to the nearest emergency department (ED) were retrieved from Statistics Norway’s databases,^24,25^ which offer detailed information at the municipality level across all Norwegian regions (please see *Data availability* section of this paper for direct links).

Furthermore, we used data from a national online survey targeting all MIPAC units.^21^ The survey collected MIPAC unit-level data on organizational characteristics, staffing, nursing competence, quality standards, and leadership. It was distributed to managers of 226 MIPAC units identified by the Norwegian Directorate of Health at the beginning of 2019.

For the survey, all first-line nurse managers in operational MIPAC units identified by the Norwegian Directorate of Health in early 2019 were invited to participate. No additional inclusion or exclusion criteria were applied at this stage. Respondents were informed about the study by telephone and invited to complete the online questionnaire, which was distributed in early March 2019. Non-respondents were contacted by telephone to ensure receipt, and one email reminder was sent before the survey closed in early June 2019. The final response rate was 92% (n = 207), yielding a rich dataset at the MIPAC unit level.

The questionnaire was developed by the research team for the University of Agder study and was designed to collect factual information about organizational structures and practices. It was not intended to measure latent constructs and, therefore, was not validated in the psychometric sense. Prior to data collection, the questionnaire was pilot tested with five individuals from the target group (unit managers), representing a small proportion of the total population, to assess clarity, logical flow, and structure. Feedback from this process led to minor adjustments to improve readability and coherence.^21^ The questionnaire is not provided as a supplementary file because it was developed in Norwegian for use in this national study and is not available in an English version. Readers interested in the questionnaire may contact the corresponding author for further details.

For the present analysis, units were included if they could be matched across all three data sources and had complete data for the variables of interest. Units were excluded if they lacked complete data or could not be linked across datasets. After merging the three sources, the analytical sample comprised 147 units, corresponding to 68% of the 215 MIPAC units included in the administrative dataset. The resulting analytical dataset contained complete data on all variables used in the regression analyses, with no missing values.

### Study Variables

The main outcome variable in the multivariate analysis was the percentage of admissions in 2019 involving patients aged 80 years and older, calculated at the MIPAC unit level. For each unit, this variable represents the share of total admissions consisting of patients in this age group. Independent variables included service location, health region, MIPAC unit size, physician and registered nurse (RN) coverage, percentage of adults aged ≥80 in the municipality, and driving distance to the nearest ED. Service location was categorized using four dummy variables: 1) nursing home, 2) GP out-of-hours service, 3) municipal intermediate care institution, and 4) other.

Health region affiliation was represented by four dummy variables corresponding to the South-East, West, Middle, and North regions. MIPAC unit size was defined as the number of beds available within each unit. Physician coverage was measured as the total number of contracted physician hours per week at the unit. RN coverage was calculated as the percentage of RNs among the total care staff.^21^ The percentage of adults aged ≥80 in the MIPAC unit catchment area was calculated from the number of residents aged 80 and over divided by the total number of residents in the municipality(ies) served by the MIPAC, based on data from Statistics Norway.^25^ Driving distance from the MIPAC service to the nearest emergency department represents the calculated travel time (in minutes) along the road network. Ferry segments were included in the road network and assigned an effective speed limit of 12.3 km/h, as specified in the National Road Database.^24^

### Statistical Analysis

Descriptive statistics were used to summarize the data. The percentage of admissions in each age group was reported as median, minimum–maximum, and interquartile range because the distributions were not normally distributed. For the sample characteristics, categorical variables were summarized as counts and percentages (n, %), and continuous variables were reported as mean (SD) and median (min–max).

Pearson correlation analyses were conducted to examine associations among the independent variables and to assess potential multicollinearity prior to regression analysis. To explore the relationship between organizational and contextual factors and the percentage of adult patients aged 80 years and older admitted to MIPAC units, a multiple linear regression analysis was performed.

To examine the contribution of different sets of variables, variables were entered into the regression model in successive blocks (Table 5). First, MIPAC-level organizational variables were included, followed by a municipality-level demographic variable (proportion of residents aged ≥80 years), and finally, regional affiliation. Model fit was evaluated by examining increases in explained variance across successive model blocks. Only the fully adjusted model is presented in the results, as the aim of the study was to assess the combined effects of all variables.

A quadratic regression model was also tested to explore the possibility of a non-linear relationship. The model yielded a slight increase in explanatory power (R^2^ increased from 0.38 to 0.43), but residual analysis did not indicate any substantial improvement in model fit. Given the modest gain and lack of clear non-linear patterns in the data, higher-order polynomial terms (e.g., cubic or beyond) were not considered. The linear model was therefore deemed sufficient for the purposes of this analysis.

All statistical tests were two-sided, and an alpha level of 0.05 was used as the conventional threshold for statistical significance. However, we recognize that this cutoff is arbitrary and should not be interpreted as a strict dichotomy between “significant” and “non-significant” results. In line with recommendations from the American Statistical Association and methodological literature, we report exact p-values and do not categorize results solely based on whether they cross the 0.05 threshold.^26,27^ Results are presented and interpreted by considering exact p-values alongside effect sizes and confidence intervals.^28^ All analyses were conducted using IBM SPSS Statistics, version 30.

### Ethical Approval

This study was conducted in accordance with the Declaration of Helsinki. The survey data included in this study were originally collected as part of a project conducted at the University of Agder. That project received ethical approval from the Faculty Ethical Committee (FEK), Faculty of Health and Sport Sciences, University of Agder, on December 17th, 2018 (reference number RITM0037110). It was further reviewed by the Data Protection Officer at the Faculty of Health and Sport Sciences, University of Agder, and reported to the Norwegian Centre for Research Data (now Sikt – the Norwegian Agency for Shared Services in Education and Research), which assessed the planned processing of personal data in accordance with applicable data protection regulations (January 28th, 2019, reference number 815471). All participants received written information about the purpose of the project conducted at the University of Agder, the procedures involved, and their legal rights, and they provided informed consent by completing the questionnaire.

This present study is a secondary analysis of previously collected survey data and is covered by the original ethical approval and by the informed consent provided by participants. No additional ethical approval was required for this study.

The registry data used in the present analysis are publicly available and were obtained from official sources; therefore, no additional ethics approval or data protection clearance was required for their use.

## Results

Table 1 shows that patients aged ≥80 years constituted the largest age group across MIPAC units, representing more than half of all adult admissions overall. The median in this group was 54.3%, but there was considerable variation between units, ranging from 3.8% to 85.2% of all adult admissions.

**Table 1.** Percentage of Admissions by Age Group Across MIPAC Units (N=147), and Variation Between Units

​	Median (minimum, maximum)	Interquartile range
18 – 66 years old	20.7 (0.0, 86.9)	18.9
67 – 79 years old	24.7 (8.3, 55.2)	10.2
80 years old and over	54.3 (3.8, 85.2)	20.0

Table 2 presents descriptive characteristics of the MIPAC units. The majority of units (56.5%) were located in nursing homes, and the South-Eastern health region represented the largest regional group (36.1%). MIPAC unit size ranged from 0.2 to 72 beds, with a median of 2 beds. Median weekly physician coverage was 14 hours. RN coverage, expressed as the percentage of total care staff, had a mean of 62.5%. The average proportion of older adults in the municipalities where the MIPAC units were located was 5.1%. Driving distance to the nearest ED ranged from 3 to 402 minutes, with a median of 40 minutes.

**Table 2.** Sample Descriptives – MIPAC Units (N=147)

​	N	%
Location		
Nursing home	83	56.5
GP out-of-hours clinic	24	16.3
Municipal intermediate care institutions	28	19.0
Other municipal facility	12	8.2
Regional Health Authorities		
South-Eastern Norway	53	36.1
Western Norway	28	19.0
Middle Norway	24	16.3
Northern Norway	42	28.6

​	Mean (SD)	Median (min-max)
MIPAC unit size (number of beds)	4.1 (7.2)	2.0 (0.2-72)
Driving distance to nearest ED (minutes)	61.5 (65.5)	40.0 (3-402)
Physician coverage (hours per week)	26.5 (35.9)	14.0 (0-168)
Registered nurse coverage (% of total care staff)	62.5 (23.0)	​
Percentage of adults age ≥80 in MIPAC catchment area	5.1 (1.3)	​

The correlation analysis revealed clear structural patterns among the independent variables. RN coverage was lower in nursing home–based units (r = −.44, p < .01) and in municipalities with a higher share of residents aged ≥80 years (r = −.26, p < .01), but higher in larger units (r = .23, p < .01) and where physician coverage was greater (r = .19, *p* < .05). Longer driving distance to the nearest ED was associated with smaller unit size (r = −.25, p < .01) and lower physician (r = −.20, *p* < .05) and RN coverage (r = −.22, p < .01). Municipal age composition aligned with service configuration: areas with more older residents were more likely to host nursing home–based units (r = .29, p < .01). Full correlation coefficients are presented in Table 3.

**Table 3.** Pearson Correlation Matrix for Independent Variables (N = 147)

	Location_Nursing home	Location_GP out-hrs clinic	Location_Intermediate care institution	Other location	South-east RHA	West RHA	Middle RHA	North RHA	MIPAC size	Physician coverage	RN coverage	Population ≥80 (%) in MIPAC catchment area	Driving distance to nearest ED
Location_nursing home	1												
Location_GP out-of-hours clinic	-.503^**^	1											
Location_Intermediate care institution	-.552^**^	-.214^**^	1										
Other location	-.340^**^	-.132	-.145	1									
South-east RHA	-.055	.013	.105	-.069	1								
West RHA	-.028	.067	-.015	-.018	-.364^**^	1							
Middle RHA	.128	-.145	-.027	.003	-.332^**^	-.214^**^	1						
North RHA	-.022	.047	-.077	.086	-.475^**^	-.307^**^	-.279^**^	1					
MIPAC size	-.304^**^	.211^*^	.114	.103	.173^*^	.047	-.077	-.162	1				
Physician coverage	-.293^**^	.209^*^	.119	.077	.182^*^	.066	-.116	-.156	.555^**^	1			
RN coverage	-.441^**^	.259^**^	.240^**^	.104	.160	.141	-.086	-.222^**^	.225^**^	.186^*^	1		
Population ≥80 (%) in MIPAC catchment area	.291^**^	-.201^*^	-.091	-.125	-.177^*^	-.187^*^	.104	.266^**^	-.332^**^	-.182^*^	-.258^**^	1	
Driving distance to nearest ED	.102	.030	-.105	-.075	-.284^**^	-.186^*^	-.070	.521^**^	-.251^**^	-.200^*^	-.221^**^	.391^**^	1

**Correlation is significant at the 0.01 level (2-tailed); *. Correlation is significant at the 0.05 level (2-tailed). ED: Emergency department, GP: General practitioner; RHA: Regional health authority, RN: Registered nurse.

Regression analysis results for the percentage of admissions aged ≥80 years are presented in Table 4. Compared to MIPAC units located in nursing homes (reference category), units located in GP out-of-hours clinics had a lower percentage of admissions in this age group. For units located in intermediate care institutions and other municipal facilities, no statistically clear differences inthe percentage of admissions involving patients aged ≥80 years were observed. With regard to regional affiliation, the model estimates did not indicate notable differences compared to the South-East region (reference).

**Table 4.** Regression Results: Associations Between Structural and Contextual Characteristics and the Percentage of Admissions Involving Patients Aged ≥80 years

​	B (SE)	95% CI	*β*	*p*
Location				
Nursing home	Ref.	Ref.	Ref.	Ref.
GP out-of-hours clinic	-17.12 (3.23)	-23.51 to -10.73	-0.41	<0.001
Intermediate care institutions	-5.69 (2.89)	-11.41 to 0.02	-0.15	0.061
Other municipal facility	-4.28 (3.99)	-12.18 to 3.62	-0.08	0.286
Regional affiliation				
South-East	Ref.	Ref.	Ref.	Ref.
West	2.87 (2.84)	-2.75 to 8.49	0.07	0.314
Middle	5.14 (3.07)	-0.94 to 11.22	0.12	0.097
North	-3.96 (2.99)	-9.88 to 1.96	-0.12	0.189
MIPAC size	0.00 (0.18)	-0.34 to 0.35	0.00	0.988
Physician coverage (hours per week)	0.00 (0.03)	-0.07 to 0.07	0.00	0.983
RN coverage (% of total care staff)	-0.12 (0.05)	-0.22 to -0.02	-0.18	0.021
Proportion of adults age ≥80 in MIPAC catchment area	1.76 (0.92)	-0.06 to 3.57	0.20	0.057
Driving distance to nearest ED (minutes)	-0.07 (0.02)	-0.11 to -0.04	-0.31	<0.001

The final model explained 38% of the variance in admissions in the ≥80 age group (R^2^ = 0.38).

Among the continuous independent variables, RN coverage was associated with a lower percentage of admissions aged ≥80 years. Driving distance to the nearest ED was also associated with a lower percentage of admissions aged ≥80 years.

Other variables, such as MIPAC unit size and physician coverage, did not demonstrate a clear association with the percentage of admissions among individuals aged ≥80 years. The estimate for the proportion of residents aged ≥80 years in the MIPAC catchment area was positive, but the confidence interval included zero.

The sequential addition of variables in the regression model showed that MIPAC-level variables (i.e., location, size, physician and RN coverage, and driving distance) explained 31% of the variance (Table 5). Adding the MIPAC catchment area variable (i.e., proportion of adults aged ≥80 in the MIPAC unit catchment area) increased the explained variance to 34%, and including regional affiliation further increased it to 38%. These results suggest that most of the explanatory power lies at the unit level, with additional contributions from contextual factors.

**Table 5.** Sequential Addition of Explanatory Variables and Changes in Explained Variance (R^2^) for MIPAC Admission Among Patients Aged ≥80 years

Block	Included variables	R^2^	Increase in R^2^	*p* value for increase in R^2^
1	MIPAC-level	0.31	–	<0.001
2	+ MIPAC catchment area level (municipality level)	0.34	0.025	0.014
3	+ Regional level (dummy variables)	0.38	0.038	0.041

## Discussion

This study examined structural and contextual variation in the use of MIPAC services for older adults (≥80 years) in Norway. Most covariates showed weak or non-significant associations with the percentage of admissions involving patients aged ≥80 years. Nevertheless, unit location, RN coverage, and driving distance to the nearest ED were associated with the percentage of admissions aged ≥80 years, while the estimate for the proportion of adults aged ≥80 years in the MIPAC catchment area was positive but uncertain. These findings align with prior descriptions of considerable organizational variation across Norwegian MIPAC services, including variation in size, staffing, and location/integration into other services.^9,17,18,29^

The negative association between RN coverage and the percentage of admissions aged ≥80 years means that units with lower RN coverage had a higher share of admissions involving older patients with acute conditions. The estimated association was modest: each percentage-point higher RN coverage was associated with a 0.12-percentage-point lower share of admissions involving patients aged ≥80 years. Nevertheless, the direction of the association may appear counterintuitive given the complexity of older patients’ care needs. Older adults often present with multimorbidity, polypharmacy, and intertwined social and functional challenges, creating what has been described as a “care complexity” phenomenon.^30^ When acutely ill, they frequently exhibit atypical symptoms, which complicates both assessment and management. Numerous studies have demonstrated that RN availability is a critical factor in achieving positive patient outcomes across healthcare settings and countries. RN-rich environments are also associated with better safety and lower patient mortality, higher job satisfaction, reduced burnout, and lower turnover rates for staff.^31-34^ From this perspective, and consistent with Vatnøy et al (2020),^9^ these findings raise questions about whether staffing and competence in units admitting a high share of older adults are aligned with the care needs of this patient group. Because hospitalization can be physically and emotionally stressful, MIPAC represents an important alternative when appropriate. Therefore, it is important to examine whether staffing and competence in these units are aligned with the acute and complex care needs of older patients.

Unit location was also associated with the percentage of admissions involving patients aged ≥80 years. Nursing home-based MIPAC units had a substantially higher share of admissions involving patients aged ≥80 years than units co-located with GP out-of-hours clinics. The adjusted difference was approximately 17 percentage points, indicating substantial variation in the age composition of admissions between the two types of units. This variation may be relevant when considering whether staffing and competence are aligned with the patient populations served by different MIPAC units. The association with unit location should be considered together with the negative association observed for RN coverage, as units with different locations may also differ in staffing and other organizational characteristics. RN coverage was lower in nursing home-based units and in catchment areas with a higher proportion of residents aged ≥80 years, but higher in GP out-of-hours clinic-based units, intermediate care institutions, larger units, and units with greater physician coverage. These correlations indicate that RN coverage varies systematically with service configuration and local demographic context. One possible explanation for the observed differences in staffing and patient age profiles is service sorting. The associations between nursing home-based location and lower RN and physician coverage raise the possibility that these units manage a different clinical case mix, potentially including patients with less complex or more stable acute conditions, than units co-located with GP out-of-hours clinics. However, this interpretation remains tentative because patient-level clinical characteristics and case mix were not available in this study. This interpretation may be consistent with differences in the organizational roles of these services: GP out-of-hours clinic-based units may be oriented towards rapid assessment and short-term stabilization, whereas nursing home-based units are integrated with long-term care settings. Evidence from reviews of intermediate care models suggests that location and integration with existing care structures are related to patient flow, case mix, and service utilization patterns.^35^

Our findings suggest the need for a closer examination of staffing models, interprofessional collaboration, and competence requirements in MIPAC units, particularly in areas where they serve as a key alternative to hospital admission for the oldest patients. Moreover, future research could examine whether the association between RN coverage and patient age profile varies according to unit location, and whether staffing patterns are aligned with population needs.

The negative association between greater distance to the nearest ED and the percentage of admissions aged ≥80 years appears counterintuitive, as one might expect that when the ED is far away, MIPAC units would be used more frequently for older patients to avoid long travel distances. A 30-minute longer driving distance was associated with an estimated 2.1-percentage-point lower share of admissions in this age group. One hypothesis is that MIPAC units located farther from EDs serve a wider age range of patients, thereby reducing the relative share of admissions involving patients aged ≥80 years. Another hypothesis is that clinicians may adopt more conservative referral practices to MIPAC services for older patients when escalation to hospital care due to clinical deterioration would involve long transfers, prioritizing direct hospital admission to minimize risk. However, the present study did not include information about clinical decision-making or referral practices, and this explanation therefore remains hypothetical. Previous research on municipal health and care services suggests that rural services may face challenges related to smaller professional milieus and recruitment challenges.^36^ We further hypothesize that these conditions may lower physician confidence in the MIPAC services’ capacity and ability to manage complex geriatric cases safely. Previous research has also identified regional differences in discharge patterns from MIPAC units,^18^ indicating that geographical variation may be relevant to both admission and discharge patterns.

Future research could examine whether the association between geographical distance and admission patterns varies according to unit capacity, staffing, or perceived competence. Studies including information about clinical decision-making and trust in MIPAC services could also investigate possible mechanisms underlying referral patterns.

The positive point estimate for the proportion of residents aged ≥80 years in the MIPAC catchment area is consistent with the possibility that local demographic structure is related to admission patterns. However, the estimate was imprecise, and the evidence for this association remains uncertain, warranting further investigation. Municipalities with a higher share of older adults may experience greater demand for age-appropriate health services, including acute intermediate care, although this could not be established in the present study. The observed correlation between municipal age composition and RN coverage suggests that organizational and contextual characteristics may be related. Future studies could examine how demographic, organizational, and geographic characteristics are related to patterns of MIPAC utilization.

Although this study is based on pre-pandemic data from 2019, the COVID-19 pandemic constitutes an important contextual factor for interpreting MIPAC utilization in the years that followed. During the pandemic, municipal health and care services operated under heightened uncertainty, preparedness, and inter-service coordination, and most MIPAC units continued to manage acute conditions, including COVID-19–related episodes, while patients requiring longer-term follow-up were transferred to nursing homes^37,38^ in line with established practice. However, systematic national reporting on MIPAC services was not conducted in 2020, and reporting completeness in subsequent years was lower than in the pre-pandemic period.^8^ This limits detailed assessment of how service organization and utilization may have been affected during the pandemic itself.

Nevertheless, available national overviews indicate that the age distribution of patients admitted to MIPAC services remained relatively stable across the years for which data exist,^8^ suggesting continuity in the core patient population. Taken together, this implies that while temporary adaptations may have occurred during the pandemic, the structural and contextual characteristics examined in this study are likely to remain relevant for understanding patterns of MIPAC utilization beyond the immediate crisis period. At the same time, the limited availability of comprehensive (post-)pandemic data highlights the value of systematic documentation when emergency situations arise, to enable future assessment of how organizational conditions shape intermediate care services under such circumstances.

### Study Limitations

This study has several limitations that should be considered when interpreting the findings. First, the cross-sectional design limits the ability to draw conclusions about cause and effect or determine the direction of the observed associations. Because the outcome and the independent variables were measured during the same study period, we cannot establish which came first. Therefore, we cannot determine whether organizational and contextual conditions contributed to differences in admission patterns, whether patterns of service use influenced how the units were organized and staffed, or whether other factors contributed to the observed associations. The findings should therefore be interpreted as associations at the MIPAC unit level rather than as evidence of causal relationships. Second, the final analytical sample included 147 of the 215 MIPAC units in the administrative dataset. Units that could not be linked across the three data sources or had incomplete data for the variables included in the analysis were excluded. As included and excluded units were not compared, systematic differences between the two groups cannot be ruled out. This may have introduced selection bias and may limit the generalizability of the findings to all MIPAC units in Norway.

Third, the regression model did not include interaction terms, which may have obscured more complex relationships between variables—such as between RN coverage and unit location. Fourth, the analysis relied on administrative data, which did not cover patient-level factors such as functional status, comorbidity, or preferences, nor fully reflected the clinical decision-making processes. These unmeasured variables could influence both admission decisions and service utilization. Consequently, the study could not examine the mechanisms underlying the observed associations, and explanations related to service sorting, referral practices, and professional confidence should be regarded as hypotheses rather than conclusions supported by the present data. Fifth, the categorization of unit location may oversimplify the diversity of service contexts, particularly in intermunicipal or hybrid models that do not fit neatly into predefined categories. Sixth, no a priori power analysis was conducted. The sample consisted of all MIPAC units with complete data on the variables analyzed, and the study was primarily exploratory rather than hypothesis-driven. The absence of a formal power calculation may limit the ability to detect smaller associations. Seventh, all variables in the present study were aggregated and analyzed at the MIPAC unit level. Because outcome data were not available at the municipality level, it was not possible to estimate intraclass correlation coefficients (ICCs) or to apply multilevel modelling to partition variance across levels. Regional affiliation was included as a contextual covariate to adjust for broader structural differences; however, the limited number of health regions (n = 4) precluded meaningful multilevel analysis at this level. Consequently, potential clustering of municipalities within MIPAC units and unmodelled contextual heterogeneity may have led to underestimated standard errors.

## Conclusion

This study examined structural and contextual factors associated with variation in the percentage of MIPAC admissions involving patients aged ≥80 years. The model explained 38% of the variance, with the most explanatory power at the unit level. MIPAC units located in nursing homes had a higher percentage of admissions in this age group compared to those co-located with GP out-of-hours clinics. RN coverage and driving distance to the nearest ED were negatively associated with the percentage of admissions involving patients aged ≥80 years. Municipalities with a higher share of residents aged ≥80 years also tended to have a higher percentage of admissions in this age group, although the evidence for this association was uncertain. Taken together, the findings of this study may have implications for policy and resource distribution, as factors relating to service configuration, staffing, and geographic accessibility were associated with differences in the use of MIPAC services among the oldest patients. These observed differences may warrant consideration in future service planning and in further research on equitable access and high-quality care for older adults across different geographic and organizational contexts. However, the cross-sectional findings do not establish that changing these characteristics would alter admission patterns. Future research could examine the mechanisms underlying these associations and how structural and contextual factors may be related to patterns of MIPAC utilization.

## Data Availability

The publicly available data used in this study were obtained from Statistics Norway and the Norwegian Directorate of Health: • Statistics Norway: Calculated distance and drive time by road to nearest emergency room (Table 12764) • Statistics Norway: Population by age and sex in municipalities, counties, and the entire country (Table 07459) • Norwegian Directorate of Health: Status for øyeblikkelig hjelp døgntilbud The survey data used in this study are not publicly available because participants did not consent to data sharing beyond the project group.
